# The evolution of nutrition in critical care: how much, how soon?

**DOI:** 10.1186/cc11505

**Published:** 2013-03-12

**Authors:** Paul E Wischmeyer

**Affiliations:** 1 Department of Anesthesiology, University of Colorado School of Medicine, 12700 E. 19th Avenue, Box 8602, RC2 P15-7120, Aurora, CO 80045, USA

## Abstract

Critical care is a very recent advance in the history of human evolution. Prior to the existence of ICU care, when the saber-tooth tiger attacked you had but a few critical hours to recover or you died. Mother Nature, and her survival of the fittest mentality, would never have favored the survival of the modern ICU patient. We now support ICU patients for weeks, or even months. During this period, patients appear to undergo phases of critical illness. A simplification of this concept would include an acute phase, a chronic phase, and a recovery phase. Given this, our nutrition care should probably be different in each phase, and targeted to address the evolution of the metabolic response to injury. For example, as insulin resistance is maximal in the acute phase of critical illness, perhaps we have evolved to benefit from a more hypocaloric, high-protein intervention to minimize muscle catabolism. In the chronic phase, and especially in the recovery phase, more aggressive calorie delivery and perhaps proanabolic therapy may be needed. As the body has evolved limited stores of some key nutrients, adequate nutrition may hinge on more than just how many calories we provide. The provision of adequate protein and other key nutrients at the right time may also be vital. This review will attempt to utilize the fundamentals of our evolution as humans and the rapidly growing body of new clinical research to answer questions about how to administer the right nutrients, in the right amounts, at the right time.

## Is it possible that we are not evolved to survive critical illness?

Emergency medicine and critical care are very recent advances in the history of human evolution. Walter Dandy at Johns Hopkins University opened the first ICU in 1926, less than 100 years ago. For many thousands of years we (and other animal species) have evolved a metabolic response to injury that does not include the ambulance coming to the rescue, whisking us off to the operating room and then being supported in an ICU for the following weeks to months after the proverbial saber-tooth tiger attacked. So what happens when the ambulance is not coming and we are left to our body's evolved survival mechanisms? Is it possible that we have not evolved the appropriate metabolic response to survive critical illness and injury? Finally, should we not consider these evolutionary forces when deciding how best to care for our critically ill patients?

There are clearly a number of evolutionary forces at play when we look at the metabolic and inflammatory response to severe injury. These include the wishes of: Mother Nature, who is only interested in the survival of the fittest; the body, who, bending to the will of Mother Nature, appears to have evolved a metabolic response that is focused on surviving for the critical hours (or days) following injury; and physicians and patients, who in most cases hope our patients will survive a lifetime and return to the physical function and quality of life they had prior to their ICU stay.

Outside the world of civilized human society and modern medicine, the drama of what happens when the 'ambulance isn't coming' is played out in nature on a daily basis - perhaps best illustrated by the recent viral Internet video 'Battle at Kruger' [1]. The tourist-captured video shows a baby water buffalo attacked by a pride of lions and dragged into a nearby river. True to Mother Nature's desire for the weak to die and the fittest to live, a massive crocodile promptly attacks the baby buffalo from behind, while still in the clutches of the lions that are attempting to pull it from the river. The prognosis for this unfortunate buffalo looks grim as the heard of adult buffalos look on in terror. We will return to the unfortunate water buffalo's fate shortly.

Of course, in modern medicine the story often unravels quite differently, but is our evolved metabolic response to injury much different from that of the unfortunate water buffalo? If not, does our conserved metabolic response help us achieve the survival and return to the preinjury quality of life that our patients and we as physicians desire? A case study that is perhaps more relevant to our experience as critical care physicians involves a patient cared for in our hospital a number of years ago (names and key identifying details have been changed to preserve anonymity).

Joshua T was a 23-year-old male who presented to a small community hospital for an elective colectomy for intractable ulcerative colitis. He was otherwise healthy and had an uncomplicated postoperative course, until postoperative day 3 when he developed a fever to 39.5°C, shortness of breath, a productive cough and an elevated white count. He was diagnosed with pneumonia and started on antibiotics. Unfortunately, 2 days later Joshua's conditioned worsened and he rapidly developed septic shock, bacteremia and disseminated intravascular coagulation. He was subsequently noted to have a rapidly expanding abdominal hematoma and was transferred to our tertiary-care university hospital. Upon arrival at our hospital the patient was taken to the operating room and the hematoma evacuated, but due to ongoing shock and edema the surgeons were unable to close his abdomen. He subsequently suffered the unfortunate and typical course of a patient following prolonged shock. He developed acute lung injury, required significant vasopressor support in his initial ICU course and, due to his preoperative steroid therapy for ulcerative colitis, required significant stress-dose steroids.

From a nutritional and metabolic perspective, Joshua also received quite poor nutrition for a prolonged period because nutrition therapy is often only an afterthought on ICU rounds in our most severely ill patients. The patient remained *nil per os *for 5 days postoperatively, as the surgical team awaited the return of bowel sounds (which we know have no predictive value regarding extent of ileus or success of enteral nutrition (EN)) [2]. He subsequently vomited at initiation of EN, due to iatrogenic ileus because of the prolonged *nil per os *status (a common occurrence in all ICUs except the burn unit, where, despite massive injury, EN with high protein delivery is routinely started successfully within hours of injury). The patient tolerated minimal amounts of EN for the next 7 days, receiving about 50% of prescribed goal calories and protein. As a result, Joshua developed a calorie debt after 14 days of -20,000 kcal. As is common in the United States, parenteral nutrition (PN) was not started to supplement his calorie and protein needs. At this point, Joshua's clinical infection and shock had improved and ventilator weaning was attempted. However, this 23-year-old male was found to be profoundly weak and unable to be liberated from the ventilator, requiring tracheostomy. Despite his prolonged course, he was discharged from the ICU to a rehabilitation unit 48 days following admission. As is so often the case, as his ICU physicians we rejoiced, congratulating ourselves on bringing another survivor successfully back from the brink.

Was Joshua really a success? Following discharge he was unable to stand, walk or dress himself. He is unable to eat or swallow food of any sort. In fact, his number one complaint post ICU discharge was that 'I can't even change the television channel with the remote control' - his hand muscles were far too weak. While in rehabilitation, Joshua works hard to try and recover any measure of quality of life and regain his physical function. Thirty-four days post ICU discharge, while participating in range-of-motion exercises, Joshua suddenly complained of chest pain and a feeling of doom. Minutes later he is found pulseless and in full cardiac arrest. Following 40 minutes of cardiopulmonary resuscitation, Joshua was declared dead. He was ultimately found to have a pulmonary embolus from an undiagnosed lower extremity venous thromboembolism. Sadly, his tearful father, who came to visit me in the ICU shortly after his death, related the end of Joshua's story to me.

How did this death happen? As an otherwise healthy 23-year-old, Joshua should have lived - right? We had cured his ulcerative colitis, his sepsis, his lung injury and he had escaped the ICU alive. What did he ultimately die of? He died of malnutrition. The same malnutrition that serves as the number one cause of death and disability worldwide (World Health Organization statistics) and continues to cause the death of millions of Third World children. I would venture to guess that many of us believe this sort of malnutrition could never be occurring under our care in the ICU. This begs the question: are we creating survivors or victims with our current ICU care? Now you may ask: 'is this a unique and isolated "sad" outcome or does malnutrition and prolonged, post-discharge poor quality of life occur routinely in ICU patients?'

Unfortunately, we know that malnutrition is very common in acutely ill patients, occurring in 30 to 50% of hospitalized patients [3-5]. This number may be higher in critically ill patients. Hospital malnutrition has been associated with an increased risk of complications, particularly in surgical patients [5,6]. Malnutrition in hospitalized patients also increases hospital costs [7] and is associated with increased long-term mortality [8]. Unfortunately, a patient's nutritional status often becomes significantly more compromised during their ICU stay (as Joshua's did). Most troubling are data showing that more than one-half of all ICU patients worldwide are significantly underfed based on the calories and protein they are prescribed to receive for the first 2 weeks of ICU care [9]. These data, from large surveys in thousands of critically ill patients from ICUs worldwide, show that we average approximately 50% of prescribed goal calories and protein for the first 14 days of ICU care.

In addition to nutrition's probable key role in survival in the ICU setting following a prolonged illness/injury, significant mortality continues to occur after critically ill patients are discharged from the hospital. Recent data reveal that more than 40% of the 6-month mortality following severe sepsis occurs after the patient has been discharged from the ICU [10]. Many of these deaths are believed to occur indirectly as a result of catabolism, loss of lean body mass, lack of adequate physical activity and, ultimately, weakness and inability to mobilize [11,12]. Further, as shown by the seminal work of Margaret Herridge and others, many patients report very poor physical function-related quality-of-life scores for a year following ICU discharge [12]. This group has also revealed that significant decreases in physical function following an ICU stay can persist for 5 years or longer after ICU discharge [13].

So perhaps Joshua's course is more typical than all of us in the ICU community would like to admit. Can we use our knowledge of the body's evolutionary conserved metabolic response to injury and combine that with the large number of new ICU nutrition delivery trials to do better for our patients and not have them follow the unfortunate footsteps of Joshua?

## Does critical illness have phases that should guide our treatment?

**Figure 1:**
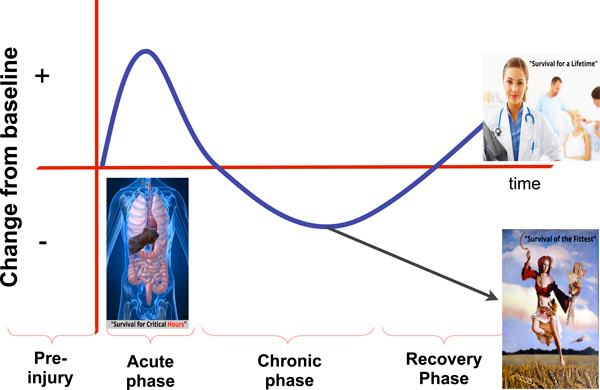
**Phased metabolic/inflammatory response to critical illness and injury**. This phased response may also be true for the hormonal, bioenergetics, and immune response. Derived from personal communication with Mervyn Singer.

It is well understood that critical illness and injury is not a single, easy to describe, homogeneous disease process. Rather, as proposed by Mervyn Singer (personal communication) and others, the body's response to critical illness occurs in phases that clearly change over time, as shown in Figure 1.

There appears to be an acute phase, consisting of the classic ebb and flow phase of shock and sepsis in which the modern ICU patient is undergoing acute resuscitation. This phase is often characterized by aggressive fluid resuscitation, vasopressor therapy, mechanical ventilation and early antibiotic therapy.

If the patient survives the acute phase, this is followed by a more chronic phase of critical illness when the patient becomes quite vulnerable to recurrent infection and other complications, which can lead to a return to the acute phase.

If the patient can recover sufficiently, they will enter a recovery phase, which often coincides with ICU discharge to a hospital floor or rehabilitation unit. During the recovery phase we hope the patient has maintained sufficient physiologic reserve to allow for recovery of quality of life and physical function sufficient to prevent a return to the chronic or acute phase of illness, or worse yet an acute and untimely death (as Joshua experienced).

The metabolic response to injury plays a unique role in all of these phases of critical illness. One could hypothesize that this metabolic response has not always evolved to lead to optimal long-term survival from prolonged ICU care.

## What is our initial metabolic response to injury and how long can it hold out?

Survival during the acute phase has always involved achieving hemostasis and preventing rapid, overwhelming infection, and quelling the systemic inflammatory response shortly after injury to prevent ongoing inflammatory injury. In this vital acute phase, the body's evolutionary metabolic response to injury is rapid catabolism. Amino acids and other key substrates are mobilized from various body storage sites, particularly muscle, to provide rapidly available energy and other key substrates for the immune system and other key organ systems as the patient (or water buffalo) fights to survive. This is the body's conserved survival system for the critical hours following injury. These substrates also appear to play a role in triggering the body's stress response system. Substrates such as glutamine, which is mobilized from muscle stores, appear to function as a signaling role to activate key stress survival proteins, such as heat shock proteins [14].

A key question concerns for how long the body's metabolic reserve can be depleted before it becomes exhausted. This answer probably depends on the patient's preinjury nutritional state and muscle mass. We know that glutamine can be depleted in the plasma as early as 48 hours after severe injury, although some patients appear able to maintain normal or even high glutamine levels for a week or more [15]. More importantly perhaps, does Mother Nature want this reserve and ultimately the severely injured patient's survival to be preserved long term? If long-term metabolic reserve was carried in the muscle, humans would have evolved to carry a more massive muscle mass if they were intended to survive severe injury that required a prolonged recovery. This larger mass may have been counter-evolutionary, as this would have made primitive man very large and slow, making him an easy target for the sharp teeth and claws of predators. Further, many early hunters literally chased their prey (say an antelope) until they died of exhaustion, and this would not have been possible with a massive muscle mass. Long-term survival following severe injury is also counter-evolutionary, because the severely injured caveman was a liability to his tribe and would not be able to gather food or reproduce; the injured man would have to be carried by his tribe, which would have led to others in the tribe being slow and obvious targets for the next passing tiger. So Mother Nature has probably favored humans and other species to evolve limited metabolic reserves, because, again, long-term survival of the severely injured does not appear to favor further evolution and survival of the species.

What does this mean for our modern ICU patients? We may need to provide more aggressive protein resuscitation or feeding in the acute and chronic phases of injury. This treatment is not to stop necessary catabolism, but to minimize it to allow the optimal chance of maintaining a physiologic and muscle function reserve that allows the patient to recover physical function and quality of life when they reach the recovery phase.

## Does our evolved metabolic response to injury impair outcome from modern surgical and trauma care by 'putting out the fire' too soon?

Of course, a key goal of the injured caveman or ICU patient in the acute phase is prevention of infection by activation of the systemic inflammatory response (SIRS). This is to provide the immune response necessary to prevent new infection following the tiger bite or to quell the inciting infection if this was the primary initiating event of the critical illness. However, the body's evolved acute phase response also needs to put out the fire almost simultaneously to prevent the SIRS response from overwhelming the host itself. Many of us would agree that in modern ICU care we rarely see a patient die of initial overwhelming bacteremia or other infection, but rather of sepsis-related multiorgan failure later in their ICU care. This is backed up by Centers for Disease Control data showing that death rates from sepsis have increased at a rate greater than any other common cause of mortality in the last year for which data were available [16] and that sepsis is now one of the top-10 causes of death in the United States [16]. As we shall see, it seems as if the early caveman's evolved immune response to the tiger bite (or the surgeon's knife) appears to be motivated to avoid committing SIRS suicide from overactivation of the inflammatory response at all costs.

**Figure 2:**
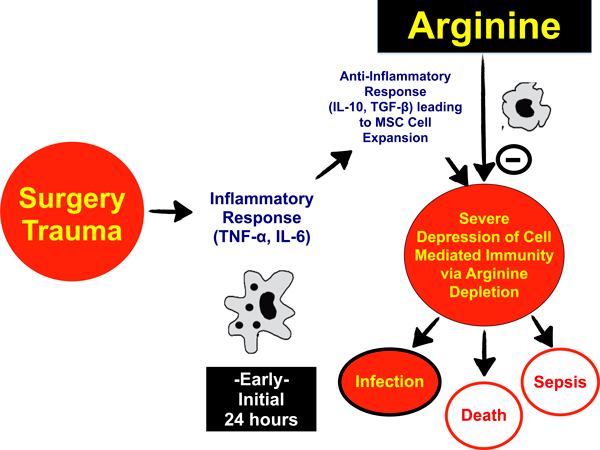
**Mechanism of immune dysfunction post surgery and trauma**. MSC, myeloid suppressor cell; TGF-β, transforming growth factor beta.

This attempt to prevent SIRS suicide is, at least in part, mediated by a well-understood metabolic process the body appears to have evolved long ago. Following the tiger bite or the surgeon's knife, there is an early initial inflammatory response (<24 hours), which is rapidly followed by apparent immunosuppression in many patients. Is it possible that the postoperative infection that ultimately led to Joshua's prolonged ICU stay and ultimate demise could have been prevented by an inexpensive nutritional intervention targeted at countering an evolutionary preserved immune-suppressive mechanism that is no longer beneficial in the modern world of surgical/trauma care? As shown in Figure 2, results of recent investigations have shown how immune function is intimately tied to arginine metabolism [17,18]. These data show that immature myeloid cells or myeloid-derived suppressor cells appear in the circulation and in lymph tissues very early post injury. Interestingly, these myeloid-derived suppressor cells express arginase-1, an enzyme that rapidly breaks down arginine. Myeloid-derived suppressor cells are known to induce a state of arginine deficiency following surgery or trauma. This deficiency is associated with suppression of T-lymphocyte and overall immune function [18]. Is it possible that supplementation with arginine can reverse the evolutionary preserved immunosuppression following injury?

Clinical outcome data, from more than 30 trials and over 3,200 patients, support this expected significant treatment benefit of arginine therapy following major surgery. In a recent meta-analysis of all these trials, arginine treatment reduced risk of infection (relative risk = 0.58; *P *<0.00001) and overall length of stay (*P *= 0.0002) versus standard EN [19]. However, very little benefit, and perhaps some harm, may be observed in septic patients [20,21]. This potential harm may be caused by arginine-mediated promotion of excessive nitric oxide production in patients with sepsis, in turn worsening SIRS and increasing risk for mortality [22].

In summary, enteral arginine therapy in the perioperative period has been given a grade A recommendation to reduce infection and shorten the length of stay compared with standard nutrition therapy. In fact, given the strength of the data from multiple trials, most experts would advocate that arginine therapy in the perioperative period should be the standard of care in high-risk surgical patients [19]. However, given that <1% of surgical patients in the United States receive arginine therapy in the perioperative period (J Ochoa, personal communication), we must do better for our patients if we hope to prevent more patients like Joshua from entering our ICUs with postoperative infections.

## What can we learn from evolution and recent clinical trials in critical care nutrition about the how, what and when of nutrition delivery in the ICU?

The basic evolutionary reason for feeding the ill or injured caveman or patient is to prevent loss of body mass and provide essential nutrients for basic biologic function. For ICU patients with a stay of 24 to 48 hours, our short-term protein and lean body mass stores are probably sufficient. However, patients that stay 3 to 7 days may lose large amounts of protein [23]. This loss is due to muscle disuse, stress/cortisol-induced catabolism, insulin resistance, and other metabolic changes. For long-stay patients, perhaps more than 7 to 10 days (like Joshua), cumulative energy and protein balance may become quite severe and have been shown to effect morbidity and mortality [24-27]

The first large evaluation of protein intake on outcome was from Alberda and colleagues [24]. This prospective observational study evaluated 2,772 ICU patients (expected to require mechanical ventilation >72 hours) from 165 ICUs around the world and found a significant inverse linear relationship between the odds of mortality and total daily calories received. The key finding of this trial was that increased amounts of calories significantly reduced mortality for patients with body mass index (BMI) <25 and BMI ≥ 35, with limited or no benefit of increased calorie intake for patients with BMI from 25 to <35. Interestingly, similar results were observed for feeding an additional 30 g protein/day. These data may indicate that nutritional reserve, particularly the lean body mass (or protein) reserve, may be vital for the effect of nutrition delivery on ICU outcome. Lean patients (low BMI <25) and obese patients (BMI >35, who may have marked sarcopenic obesity) are perhaps the patients lacking sufficient lean body mass/protein reserves to optimally survive a prolonged ICU stay without more aggressive nutrition and protein provision. A number of recent trials (that is, the EDEN trial, Arabi and colleagues' trial, and the EPaNIC trial [28-30]) have investigated the role of additional nonprotein calorie delivery on outcome. All of these trials delivered between 0.6 and 0.8 g protein/kg/day, which, as will be discussed subsequently, is far below current worldwide critical care nutrition guidelines.

## Adequate protein delivery: the key to optimal nutrition delivery in the ICU?

The ESPEN guidelines for nutrition support in the critically ill patient recommend a protein delivery of 1.3 to 1.5 g/kg/day for optimal outcomes [31] (grade B recommendation) and the ASPEN guidelines suggest 1.2 to 2.0 g/kg/day [32]. The results of trials such as the EPaNIC trial [29] revealed significant protein delivery deficits in both early-PN and late-PN groups. This deficit was due to the utilization of a low-protein PN solution, which limited protein delivery to a median of 0.8 g/kg/day throughout the trial period (even for as long as 15 days in the ICU). This trial also appeared to deliver a maximum of ~1.0 g/kg/day to any patient.

As was suggested by Alberda and colleagues [24], a small increase in protein delivery (20 to 30 g/day) improving outcome may also be supported by the recently reported OMEGA trial [33]. The OMEGA trial had a primary aim to study the effects of eicosapentaenoic acid/γ-linolenic acid/antioxidants on clinical outcomes in ICU patients with acute respiratory distress syndrome. Although no clinical effect of the eicosapentaenoic acid/γ-linolenic acid/antioxidants on mortality or other clinical outcomes was observed, there was a presently unexplained significant mortality benefit in patients receiving the control treatment. This group experienced one of the lowest mortality rates ever recorded in an acute respiratory distress syndrome trial. It is interesting to note the eicosapentaenoic acid/γ-linolenic acid/antioxidant-treated group had similar mortality to the previously reported optimal mortality for acute respiratory distress syndrome in the FACCT trial (mortality: OMEGA, 26.6% vs. FACCT (conservative fluid group), 25.5%). The observed mortality in the control formula from the OMEGA trial was a surprising 16.3%. A possible explanation for this mortality benefit lies in the realization that the control formula given in the OMEGA trial delivered over five times the amount of protein per day to patients in the higher survival control group (20 g protein/day from control supplement vs. 3.8 g protein/day from study supplement). Although this is only a hypothesis and the OMEGA trial was not designed to detect this possible effect, the protein delivery difference achieved almost approximates the 30 g protein/day shown to reduce mortality in ICU patients by Alberda and colleagues. These data taken in concert with data from the recent trials by Weijs and colleagues and Allingstrup and colleagues [34,35] support the hypothesis that additional protein (approximately 30 g/day depending on patient weight) may be beneficial in reducing the mortality hazard risk. Finally, initial data from the large REDOXs trial indicate that patients receiving an additional 30 g protein/day had improved Short Form-36 physical function scores at 3 months post discharge (D Heyland, personal communication).

Large randomized controlled trials examining the effect of nutrition delivery with adequate protein delivery (1.2 to 2.0 g/kg/day) on outcome are currently needed, as these data do not currently exist.

## Can we make practical recommendations (or at least a hypothesis) for what, when and how from evolution and clinical data for a phased approach to nutrition in the ICU?

Based on what we have learned from Mother Nature, the evolution of the body's metabolic response in the critical hours, and our desires for our patients as ICU physicians, we can synthesize the following phased approach to nutrition in the ICU patient. This phased approach attempts to synthesize the latest clinical data with evolution to attempt to provide a glimpse of the future of critical care nutrition.

**Figure 3:**
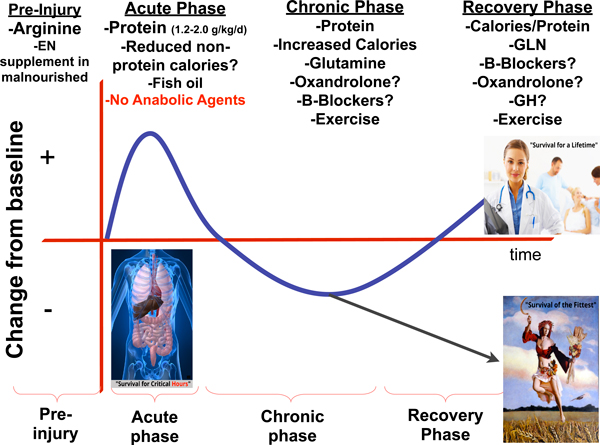
**Phased-based approach to nutrition delivery in critical care**. EN, enteral nutrition; GH, growth hormone; GLN, γ**-**linolenic acid.

As shown in Figure 3, we can take our phases of critical illness and add a preinjury phase during which preoperative optimization can be initiated to attempt to improve patient outcomes. This figure summarizes focused nutrition interventions that the literature currently supports, and where evidence is unclear proposes an evolutionarily based hypothesis.

The preinjury phase is a phase where nutrition and metabolic status can be initiated to optimize outcomes postoperatively. Intervention with arginine nutritional formulas can reduce infection and shorten the length of hospital stay [36]. Further, preoperative EN or PN supplementation in malnourished patients has clearly been shown to improve outcome following high-risk surgeries and is a grade A recommendation of current societal nutrition guidelines [37].

Based on recent clinical trials, basic physiology and evolution, the acute phase may be a period when a reduced-nonprotein-calorie, high-protein delivery (1.5 to 2.0 g/kg/day) may be optimal. One could argue that during critical illness, particularly in well-nourished patients (BMI 25 to 35), sufficient nonprotein energy substrates are available from endogenous sources for some period of time after the onset of illness. Importantly, measurements of early sepsis energy expenditure in the acute phase have shown a negative correlation of energy expenditure (or reduced energy expenditure) with increasing severity of sepsis [38]. Energy expenditure is then found to increase significantly in the more chronic phase of sepsis or critical illness [39]. However, significant breakdown of protein stores early in the ICU stay indicates they are being mobilized for energy metabolism and other key cellular functions. In the early period there may be little to counteract protein loss from the body, other than protein or amino acid supply from outside by EN or PN supply. In Weijs and colleagues' recent trial, the group with adequate protein but insufficient energy supply led to the lowest mortality rate of any studied group, even lower than the group with adequate energy and protein supply [35]. The hypothesis of aggressive, early nonprotein calorie delivery being detrimental or at least not beneficial in the ICU is perhaps best exemplified by the results of the EpaNIC trial [29]. This trial utilized aggressive PN glucose loading in the early PN group via a low-protein PN product, leading to a significant nonprotein calorie load (with low protein delivery) versus the late-PN group. This trial showed significantly better ICU outcomes in patients who did not receive this early, aggressive nonprotein calorie load.

In the chronic phase of critical illness, energy expenditure as measured by calorimetry increases significantly [39] and thus increased nonprotein calories should be delivered and sufficient protein (1.5 to 2.0 g/kg/day) should continue to be given. In this phase, administration of glutamine has been shown to reduce mortality in critically patients requiring PN and should be given to patients receiving PN in this time period [31]. In this phase of critical illness, as the SIRS response begins to subside, anabolic agents such as oxandrolone or anti-catabolic agents such as propranolol could possibly be considered to reduce ongoing, potentially futile, hyper-metabolism and to induce recovery of lean body mass. It is key that any use of potent anabolic therapies is given with adequate nutrition delivery (as is achieved in virtually all burn patients) to provide the building blocks for anabolic processes. Anabolic therapy in the absence of adequate energy and protein delivery is likely to be detrimental. This strategy has been used to improve outcome following burn injury and is receiving ongoing study in that realm [40]. Initiation of early mobility or physical therapy programs in patients in this phase of illness are beginning to show benefit on long-term functional outcomes - as demonstrated recently by Kress and colleagues, who demonstrated benefit of early physical and occupational therapy in ventilated patients [41].

In the recovery phase, when C-reactive protein and other markers of inflammation are often much decreased, continued protein and calories are required to continue the recovery of lean body mass and physical function required for independent living and quality of life. In this period, perhaps stronger consideration should be given to anabolic agents such as oxandrolone. Perhaps even the topic of growth hormone therapy should be revisited, as has been suggested recently by Taylor and Buchman [42]. If growth hormone therapy was to be studied in this period, a much-reduced dose should be utilized versus those that have been studied in previous trials of critical illness and adequate protein and calorie delivery must be ensured to provide the key substrates for anabolism [43]. Ongoing trials of aggressive physical therapy in the recovery period post ICU continue and results are anxiously awaited.

## Conclusion: can we help defeat Mother Nature and help the least fit to survive?

As we think about the future of critical care, and specifically critical care nutrition, the concept of phases of critical illness and optimal delivery of nutrition in the ICU creates a great deal of research questions that need to be answered. These questions include 'How do we define the transitions from the acute phase to the chronic phase and then to the recovery phase?' and 'How do we define when a patient has relapsed back to the acute phase?' It is possible that one or (more probably) a combination of biomarkers (IL-6, procalcitonin, C-reactive protein, mitochondrial markers of metabolic hibernation, and so forth) may be able to assist in defining rough transition points in these phases of illness. Other key questions needing to be answered include the possibility in well-nourished patients (BMI of 25 to 35?) that a reduced nonprotein calorie delivery (hypocaloric, high protein) coupled with adequate protein delivery early in ICU care (acute phase) may be optimal. More importantly, large trials examining the basic effect of nutrition delivery with adequate protein delivery (1.2 to 2.0 g/kg/day) on outcome are needed, as these data do not currently exist. Finally, better methods are needed by which to evaluate the patient's admission nutrition status and lean body mass throughout ICU care. These include easily accessible bedside methods such as the ultrasound lean body mass technique currently being tested in the TOP-UP trial of supplemental PN [44]. Improved nutrition evaluation methods may finally allow us to better target patients at risk for malnutrition and reduced lean body mass so we may provide more aggressive nutrition delivery to those who are the most nutritionally at risk.

In closing, I believe it is clear that we as humans are not optimally metabolically evolved to survive modern, prolonged ICU care. Mother Nature and her survival of the fittest mentality just would not allow for it. However, as demonstrated by the eventual fate of the all-but doomed baby water buffalo in 'The Battle at Kruger' [1], if we work together with continued clinical trials and clinical care targeted to specific patient needs, delivering the right nutrients, at the right time, in the right amounts, perhaps even the least-fit patients can survive, and in the future patients like Joshua will have a different outcome.

## Abbreviations

BMI: body mass index; EN: enteral nutrition; IL: interleukin; PN: parenteral nutrition; SIRS: systemic inflammatory response.

## Competing interests

PEW is an occasional consultant and speaker for Fresenius, Baxter, Nestle and Abbott.
